# Editorial: Nutrition and sustainable development goal 8: decent work and economic growth

**DOI:** 10.3389/fnut.2024.1500304

**Published:** 2024-12-02

**Authors:** Romanus Osabohien

**Affiliations:** ^1^Institute of Energy Policy and Research (IEPRe), Universiti Tenaga Nasional (UNITEN), Kajang, Malaysia; ^2^DEPECOS Institutions and Development Research Centre (DIaDeRC), Ota, Nigeria

**Keywords:** sustainable development, nutrition and sustainable development, food and nutrition security, decent work and economic growth, SGD-8

## Introduction

The United Nations' Sustainable Development Goal 8 (SDG-8) emphasizes the promotion of inclusive and sustainable economic growth, full and productive employment and decent work for all. At the core of this objective is the recognition that human capital—physical and cognitive abilities—is a critical driver of economic performance. Nutrition plays a fundamental role in shaping human capital and by extension, influences economic productivity, employment outcomes, and societal wellbeing (1, 2). The Research Topic, “*Nutrition and Sustainable Development Goal 8: Decent Work and Economic Growth*,” brings together research that illustrates the intricate connections between nutrition and the ability of individuals and communities to participate in and benefit from economic activities.

This editorial aims to reflect on the key contributions made in this Research Topic, demonstrating how nutrition not only influences individual health and wellbeing but also underpins economic performance and decent work. Through a synthesis of the five published articles, this editorial explores the common themes, challenges, and opportunities presented by the research in this Research Topic.

## Overview of published articles

The articles published in this Research Topic investigate the relationship between nutrition and economic growth through various lenses, ranging from childhood stunting and maternal employment to dietary patterns among healthcare professionals. Each study contributes to a broader understanding of how nutrition shapes human capabilities and productivity in different socio-economic contexts.

Tekeba et al. explore disparities in stunting among preschool children of employed and unemployed mothers in Gondar City, Ethiopia. Their comparative cross-sectional study underscores the importance of maternal employment status in determining nutritional outcomes for children. The findings indicate that children of unemployed mothers are more likely to experience stunting, which could have long-term implications for educational attainment and workforce participation. Wang et al. apply a deep neural network model to predict diet quality among healthcare workers in North China during the COVID-19 pandemic. The study highlights how high-stress professions, such as those in healthcare, influence dietary patterns, which in turn affect workforce productivity and wellbeing. The use of machine learning in this context opens new pathways for real-time monitoring and interventions aimed at improving diet quality among essential workers.

de Rijk et al. investigate how macronutrient intake affects alertness during night shifts, a critical factor for productivity and safety in industries reliant on shift work. Their findings emphasize the timing of nutrient intake as a key determinant of cognitive function and alertness, suggesting that better-designed meal plans could enhance worker efficiency in sectors where night shifts are common.

Dohms et al. focus on the quality of food preparations offered to workers in different food service settings. Using a qualitative assessment score, the study compares nutritional quality across workplaces, demonstrating that many employees lack access to healthy, nutrient-rich meals. This highlights a major opportunity for employers to improve worker health and productivity by investing in better food services.

Abdulahi et al. explore how maternal socio-economic status determines the consumption of animal-source foods among children in East African countries. The study's multilevel mixed effects model reveals that children from higher socio-economic backgrounds consume more nutrient-dense foods, a disparity that exacerbates inequality in health and future economic potential. This underscores the importance of addressing socio-economic barriers to ensure equitable access to nutrition, particularly in early childhood.

## Key themes and insights

The studies in this Research Topic collectively point to several critical themes at the intersection of nutrition, employment, and economic growth:

### Employment and nutritional outcomes

Tekeba et al.'s work emphasizes how employment status, particularly for women, plays a vital role in the nutritional wellbeing of families. This highlights the need for policies that not only promote maternal employment but also provide support systems—such as childcare and nutrition programs—to ensure that employment translates into improved nutritional outcomes for children.

### Workplace nutrition and productivity

Wang et al. and de Rijk et al. explore how workplace conditions, including job stress and shift work, influence dietary patterns and, by extension, productivity. These studies demonstrate that nutrition is not only a matter of personal health but also a strategic asset for enhancing workplace performance. Employers, therefore, have a stake in ensuring that workers have access to nutritious meals, especially in high-stress and irregular working conditions.

### Socio-economic status and nutritional inequality

Abdulahi et al.'s findings bring to light the enduring disparities in nutrition based on socio-economic status. Access to nutrient-rich, animal-source foods remains a privilege of the wealthy, with long-term implications for child development and future workforce capabilities. Tackling these inequalities is essential for creating a more inclusive economic growth model where all children have the nutritional foundation to succeed.

### Interventions for nutritional equity

Dohms et al. suggest that improving food quality in workplaces can have a direct impact on workers' health and economic output. By focusing on nutritional interventions within workplace food services, employers can play a role in bridging the nutritional gap for lower-income workers who might otherwise lack access to healthy meals.

## Challenges and opportunities

While the research presented in this Research Topic illuminates the vital role of nutrition in economic growth and employment outcomes, it also highlights several challenges. Socio-economic disparities continue to undermine efforts to ensure equitable access to nutrition, particularly in lower-income countries. The studies also indicate that workplace environments, especially for those in high-stress or low-income jobs, do not always provide the nutritional support needed to maintain productivity and wellbeing.

Furthermore, the findings also point to significant opportunities. Policymakers can leverage these insights to design interventions that target vulnerable populations, such as children in low-income households and workers in stressful or irregular occupations. Workplace nutrition programs, child nutrition initiatives, and efforts to improve access to animal-source foods are all critical interventions that can promote both decent work and sustained economic growth.

## Conclusion

This Research Topic highlights the multifaceted relationship between nutrition, decent work and economic growth. From childhood stunting to the dietary challenges faced by healthcare workers and shift workers, the research emphasizes that nutrition is a key determinant of both individual wellbeing and economic productivity. Achieving SDG-8 requires a holistic approach that integrates nutrition into strategies for promoting decent work and economic growth.

Looking forward, future research should continue to explore how nutrition can be harnessed as a tool for promoting inclusive economic development. Interdisciplinary collaboration between economists, nutritionists, and public health experts will be essential for identifying and implementing effective interventions. By ensuring that nutrition is prioritized in policies aimed at fostering economic growth, we can make meaningful progress toward achieving both SDG 8 and broader sustainable development goals.
